# Nasal Cytology: A Comparative Study of Two Different Techniques of Processing—Smeared versus Cytocentrifuged Slides

**DOI:** 10.1155/2018/1640180

**Published:** 2018-11-14

**Authors:** Maria Laura Bartoli, Lodovica Cristofani-Mencacci, Mariella Scarano, Andrea Nacci, Manuela Latorre, Elena Bacci, Pierluigi Paggiaro, Veronica Seccia

**Affiliations:** ^1^Cardio-Thoracic and Vascular Department, University of Pisa, Via Paradisa 2, 56124 Pisa, Italy; ^2^1st Otorhinolaryngology Unit, Department of Neuroscience, Azienda Ospedaliero Universitaria Pisana, Via Paradisa 2, 56124 Pisa, Italy; ^3^ENT, Audiology and Phoniatric Unit, Department of Neurosciences, University of Pisa, Via Paradisa 2, 56124 Pisa, Italy

## Abstract

Nasal cytology is a precious tool to study nasal disorders, but in current literature, there is no consensus on the standardization of the processing procedure of the obtained samples. Therefore, we decided to test on specimens obtained by nasal scraping, a common way of nasal specimen sampling, two different processing techniques, smear and cytocentrifugation, and compare them in terms of inflammatory cell content, quality of slides, and validity on clinical assessment. We analyzed 105 patients with suspected sinonasal diseases, and in each patient, we performed nasal cytology with both techniques. Our analysis showed a good correlation between the two techniques for neutrophil and eosinophil percentages, both returned well-preserved cells, and showed higher neutrophil percentage in males and in smokers and higher eosinophil percentage in patients with polyposis, with a good concordance with clinical symptoms, as measured by a specific disease-related questionnaire (Sino-Nasal Outcome Test-22). Technically speaking, smeared slides were easier to prepare, with no need of dedicated equipment, but cell distribution was better in cytocentrifuged slides allowing shorter reading time. In conclusion, both techniques can be considered superimposable and worthy to be used.

## 1. Introduction

Chronic rhinitis is a very common condition throughout the world, with up to 20 million patients affected by nonallergic rhinitis (NAR) in the United States and 50 million in Europe [1]. Great importance is nowadays given to this condition, not only for its economic burdens [2] but also for its impact on Quality of Life (QoL): in fact, chronic rhinitis is associated with poorer job or school performance and it may interfere with sleep, intellectual functioning, and recreational activities.

From a clinical point of view, chronic rhinitis is defined as the presence of at least one of the following: congestion, rhinorrhea, sneezing, nasal itching, and nasal obstruction, with (AR) or without (NAR) a concordance between symptoms and allergen seasonality [3]. AR and NAR may coexist in the same patient, resulting in the diagnosis of mixed rhinitis (MR) [4] or overlapped rhinitis [5]. NAR can be further classified in different subtypes on the basis of cytological criteria, in accordance with prevalent inflammatory cellular population at nasal cytology (with eosinophils: NARES; with neutrophils: NARNE; with eosinophils and mast cells: NARESMA; with only mast cells: NARMA) [3, 6] and/or according to clinical characteristics (e.g., senile, gustatory, or atrophic) [3, 7]. Nasal inflammation is also frequently reported in systemic disorders, like EGPA [8] or rheumatologic conditions [9].

Therefore, nowadays, nasal cytology has acquired an increasing role in the diagnosis and management of NAR and MR and its current day-by-day use is recommended by some authors [6].

However, up to now, there is no consensus on how to sample and process the nasal specimen. Different techniques for sampling are described in literature, such as nasal wash, blown secretions, nasal brushing, or scraping [10]. Among them, the most frequently adopted are the scraping and brushing methods, which are easy to perform and painless for the patient. The collected material may be processed by direct glass smearing or by dilution of the samples with phosphate-buffered solution (PBS) or dithiothreitol (DTT) coupled with cytocentrifugation [6, 11, 12]. Quantitative and semiquantitative evaluations are reported [6, 12].

This study is aimed at performing on samples obtained by nasal scraping, a direct comparison between smearing and cytocentrifugation preparing techniques, in terms of inflammatory cell content, quality of preparation, and validity on clinical conclusions. At the best of our knowledge, no previous data on this comparison have been reported.

## 2. Materials and Methods

### 2.1. Patients

Patients were recruited in the Rhinologic Outpatient Clinic of the 1st Otorhinolaryngology Unit (Pisa University Hospital) in a period between January 2013 and July 2014. All patients gave a written informed consent, as part of the clinical routine.

For all patients, we performed a detailed personal history, a recording of the medications used, the allergic profile, and the possible coexisting comorbidities (e.g., asthma, eosinophilic granulomatosis with polyangiitis, and Sjögren syndrome), followed by a fiberoptic nasal endoscopy.

The possible diagnoses for sinonasal district were (a) normal, in cases of no nasal symptoms and no sinonasal endoscopic alterations; (b) rhinitis, diagnosed on a clinical basis, considered allergic (AR) if at least one skin test result was positive and there was a concordance with the chronological pattern of symptoms; if not, rhinitis was defined as “nonallergic” (NAR) and further classified with nasal cytology accordingly to the predominant inflammatory cellular population [6]. Duration and severity of both AR and NAR were classified according to Allergic Rhinitis and its Impact on Asthma (ARIA) recommendations [13]; (c) rhinitis sicca, in patients referring the subjective sensation of “dry nose,” coupled with visible dry nasal mucosa and atrophic nasal turbinates and Sjögren's syndrome [14]; (d) chronic rhinosinusitis with (CRSwNP) and without (CRSsNP) nasal polyps, in cases of nasal blockage, nasal drip, facial pain, reduction of smell, associated to endoscopic nasal signs of polyps, mucopurulent discharge, and/or mucosal edema, according to European Position Paper on Rhinosinusitis and Nasal Polyps [15]. All patients fulfilled a health-related questionnaire focused on sinonasal disorders, the Sino-Nasal Outcome Test-22 (SNOT-22) [16], as a part of our routine clinical protocol.

### 2.2. Specimen Collection and Analysis

At the end of the clinical examination, according to the methods described in literature [6, 15] and currently adopted in our center, two consecutive nasal scrapings were performed collecting the material from the middle third of the inferior turbinate by means of a Rhino-Probe™ curette (Arlington Scientific Inc. Springville, Utah, USA). Treatments, if any, were discontinued before nasal scrapings. The withdrawal period was at least 4 days for antihistamines and 10 days for topical steroids [17]. One curette was immediately smeared on a glass slide paying attention to properly distribute the collected material on the slide and to dissipate the possible clots of mucus. The other curette was placed in a 15 ml polypropylene tube with 0.5 ml of PBS, left in a 37°C shaking bath for 10′ to let cells resuspend in the PBS solution. The suspension was then cytocentrifuged (Cytospin II, Shandon Scientific, Sewickley, PA, USA) at 500 rpm for 5 minutes in order to obtain a monolayer of cells onto a defined area of the slide. After air-drying, both smeared and cytocentrifuged slides were fixed and stained using Diff Quik (Baxter Scientific Products, Miami, FL). Slides were examined blindly by two different readers. For each slide, at least 300 inflammatory cells or 30 fields at 40x magnification were examined by light microscopy (Olympus BH2, Tokyo, Japan). Lymphocytes, neutrophils, eosinophils, mast cells, and epithelial cells were counted and expressed as percentage of total cells. According to a recent publication [6], a semiquantitative evaluation of slides, using as inflammatory cell grading (none, occasional, few, moderate number, large clumps, and clumps covering the field), was also performed. Only eosinophils and neutrophils, the most representative inflammatory cells, were used in the statistical analysis to compare cell differentials between the two techniques.

### 2.3. Statistical Analysis

Cell percentages are expressed as median (range). Differences among groups were tested using ANOVA and Mann–Whitney test for normally and nonnormally distributed variables, respectively. Cell percentage differences among the different classes of rhinitis were analyzed by the Kruskal-Wallis test. A *p* value lower than 0.05 was considered as significant. Intraclass correlation coefficients (RI) were calculated to assess the concordance between the two techniques, when inflammatory cells were expressed in percentage, and to evaluate the concordance between the two readers, assessed on 20 slides randomly selected for each technique. RI values > 0.6 were considered as satisfactory [18]. Concordance between the two techniques for the semiquantitative evaluation of inflammatory cells was calculated using Cohen's kappa coefficient. *K* values > 0.6 were considered as satisfactory [19]. For both techniques, the concordance between readers was good (RI = 0.84 for neutrophils and 0.96 for eosinophils in cytocentrifuged slides and RI = 0.80 for neutrophils and 0.89 for eosinophils in smeared slides). Statistical analysis was performed using the SPSS 16.0 statistical program.

## 3. Results

120 subjects were initially enrolled in our study, but 15 of them were successively excluded because of insufficient or damaged cells in smeared (*n*: 9) or cytocentrifuged (*n*: 6) slides. Therefore, the comparative analysis was performed on 105 patients, whose data are reported in Table 1. Patients were fairly well distributed in the different categories of rinosinusal disease, with a prevalence of patients with CRSwNP. SNOT-22 score was always higher in patients than in controls, with the highest values observed in AR and CRSwNP.

All patients underwent a double sampling of the mucosa of the inferior turbinate, and no side effects, namely, epistaxis, were observed.

**Figure 1:**
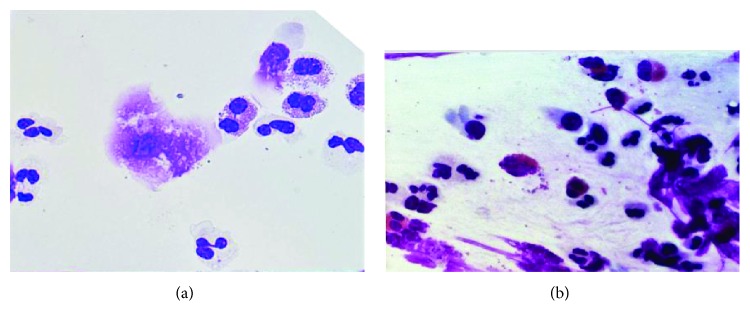
Examples of cytocentrifuged (a) and smeared (b) slides.

Cells were well preserved both in smeared and cytocentrifuged slides. Cell distribution was better in cytocentrifuged slides, with well-separated and easily identifiable cells. Smeared slides showed more clumps and areas covered with mucous film (Figure 1).

**Figure 2:**
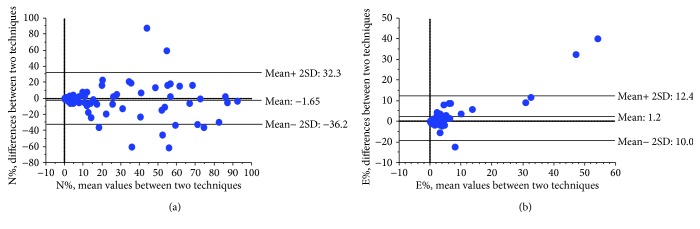
Bland-Altman plot of difference against neutrophil (a) and eosinophil (b) means (in % values) obtained with the two different processing techniques.

The two methods showed a good correlation for neutrophil (8.0 (0–91)% vs 10.2 (0–97)%, rho: 0.8; *p* < 0.0001, cytocentrifuged vs smeared slides) and eosinophil percentages (0.2 (0–74)% vs 0.2 (0–34)%, rho: 0.76; *p* < 0.0001, cytocentrifuged vs smeared slides), with high value of RI for both cell types (Figure 2).

Only at high eosinophil percentage level, the cytocentrifugation preparing technique tended to show higher values. A good agreement was also observed performing on the same cell population a semiquantitative analysis (Table 2).

We observed higher neutrophil percentages in males vs females, in smokers and former smokers in comparison with nonsmokers, and higher eosinophil percentages in patients with nasal polyps, both in cytocentrifuged and smeared slides (Table 3).

We also found a positive correlation between eosinophil percentages and SNOT-22 score with both methods (*p* < 0.008, rho: 0.4, both in smeared and cytocentrifuged slides). This observation was confirmed in the semiquantitative evaluation by the significant increase of SNOT-22 score from the none to the very large number of eosinophils classes (*p* < 0.01 in both smeared and cytocentrifuged slides).

## 4. Discussion

Nasal cytology was firstly applied in the clinical practice at the beginning of the twentieth century, when Eyermann identified some eosinophils in the nasal mucosa of allergic patients [20], but only much later, in the 1970s in a random manner [21], and more systematically from the 2000s [22], nasal cytology found its role in nasal diagnostic algorithm. The successive wide application of nasal cytology in rhinology derives from the simplicity of normal nasal mucosa structure, which is formed by a ciliated pseudostratified epithelium, composed of mucosecreting cells and ciliated, striated, and basal cells [6]. Therefore, on a rhinocytogram, the presence of inflammatory cells or infectious pathogens (biofilm, bacteria, and fungi) is pathologic and a marker of nasal disease [23, 24].

The sampling procedure is painless and minimally invasive. The cell count together with the clinical and allergic profile of the patient allows the clinician to formulate a proper subdiagnosis in the field of nonallergic rhinitis (NAR), individuating NAR with eosinophil prevalence (NARES), with neutrophil predominance (NARNE), with mast cells (NARMA), and with eosinophils and mast cells (NARESMA) [25], and can identify MR [4]. More recently, nasal cytology was used as a tool to evaluate the response to treatment and to prospectively provide a prognostic score of relapse of nasal polyps in patients undergoing sinonasal surgery for CRSwNP [26].

Despite the increasing application of nasal cytology in the diagnosis of sinonasal disorders, only few methodological studies have been published. To our knowledge, this is the first study comparing two different methods of processing nasal scraping that we accepted as the best sampling technique because it is considered the most common way to collect nasal material. We compared smearing and cytocentrifugation preparing techniques in the same patients.

The two techniques returned similar results both in terms of diagnosis support and classification of patients according to sex, smoking habit, and presence of nasal polyps. The semiquantitative counting method concordance was acceptable, and also, the correlation for neutrophil and eosinophil percentages between the two techniques of processing was high. In this attempt, two methods of processing resulted completely similar in terms of repeatability and validity.

We found that cells were well preserved in both smeared and cytocentrifuged slides. Preparation steps were easier and quicker with the smearing technique performed without the need of extra instrumentation, while cytocentrifuged slides preparation was more time consuming, requiring an equipped laboratory. On the other hand, cytocentrifuged slides were easier to read, having a better and more homogeneous distribution of cells in comparison with smeared slides, where the cells were often concentrated in clots and hardly recognizable, thus returning in an analysis sometimes difficult. This resulted in a great reduction of cytocentrifuged slides reading time.

At the present time, nasal cytology is seldom used in the assessment and management of patients with upper airway diseases, due in part to the time-consuming procedure, the conflicting reproducibility of the results, and the still moderate interest in phenoendotyping chronic upper airway diseases. However, there is now a growing interest in the definition of specific inflammatory patterns in the upper and lower airway diseases, which are potentially useful for a better management of these diseases according to the so-called “precision medicine” [27]. This new era is currently applied in the identification of different endotypes of severe asthma, and it could be relevant also for the management of chronic rhinosinusitis. In this attempt, allergologists, pulmonologists (mainly asthmologists), and ENT specialists should become always more familiar with this tool for assessing the characteristics of upper airway inflammation. If nowadays nasal cytology is mainly limited to research centers, in the next future, it might be more extensively applied in the context of a “personalized medicine.”

On the basis of our results, we can conclude that there is an overall overlap of the data deriving from smears and cytocentrifuged slides. Although the cytocentrifuged slides allow to obtain a better quality of slides, thus making simpler and less time consuming the reading procedure, this advantage is counterbalanced by a more complex and time-consuming preparation procedure. Therefore, both procedures might be considered as equivalent in terms of clinical usefulness.

This study could significantly improve the diffusion of nasal cytology in the assessment of upper airway diseases.

## Figures and Tables

**Table 1:** Demographics of patients.

	Number (%)	Sex, males (%)	Age, years	Smoke, yes/ex (%)	SNOT-22
All patients	105	39 (37.1%)	57.4 ± 13	25 (23.8%)	38.6 ± 20.0
Normal controls	10 (9.5%)	2 (20.0%)	61.3 ± 11.9	2 (20.0%)	21.5 ± 10.7
AR	17 (16.2%)	6 (35.3%)	58.4 ± 13.6	3 (17.6%)	48.3 ± 20.1
NARNE/NARES	18 (17.1%)	7 (38.9%)	48.5 ± 16.7	3 (16.7%)	40.6 ± 9.9
Rhinitis sicca	14 (13.3%)	0	63.1 ± 10.9	3 (21.4%)	34.3 ± 12.2
CRSwNP	30 (28.6%)	13 (43.3%)	58.9 ± 12.6	8 (26.7%)	42.3 ± 22.9
CRSsNP	16 (15.2%)	11 (68.8%)	57.3 ± 12.1	6 (37.5%)	34.2 ± 24.5

AR: allergic rhinitis; NAR: nonallergic rhinitis; NARNE: nonallergic rhinitis with neutrophils; NARES: nonallergic rhinitis with eosinophils; CRSWNP: chronic rhinosinusitis with polyps; CRSsNP: chronic rhinosinusitis without polyps.

**Table 2:** Inflammatory cell count in smeared (SS) and cytocentrifuged (CS) slides. Data are expressed as number and percentages (%) of samples within each inflammatory cell category.

Cells	Neutrophils in CS	Neutrophils in SS	Eosinophils in CS	Eosinophils in SS
*N*: 105	*N* (%)	*N* (%)	*N* (%)	*N* (%)
None	26 (24.8)	18 (17.1)	58 (55.2)	60 (56.2)
Occasional	16 (15.2)	14 (13.3)	22 (21.0)	22 (21.0)
Few	17 (16.2)	22 (21.0)	11 (10.5)	14 (13.3)
Moderate	13 (12.4)	16 (15.2)	10 (9.5)	5 (4.8)
Large number	2 (1.9)	2 (1.9)	0	0
Very large number	31 (29.5)	33 (31.4)	4 (3.8)	4 (3.8)

*K* Cohen	0.699		0.724	
*p*	<0.001		<0.001	

CS: cytocentrifuged slides; SS: smeared slides.

**Table 3:** Distribution of inflammatory cell percentages in smeared (SS) and cytocentrifuged (CS) slides according to sex, smoking habit, and presence of nasal polyps. Mann–Whitney statistical test was used to evaluate differences between groups.

	Neutrophils (%), in CS	Neutrophils (%), in SS

Males	13.4 (0–87)	16.0 (0–97)
Females	1.1 (0–91)	2.1 (0–94)

	*p* = 0.003	*p* = 0.003

Smokers/ex-smokers	8.7 (0–91)	13.0 (0–97)
Nonsmokers	1.2 (0–86)	3.1 (0–87)

	*p* < 0.001	*p* = 0.03

	Eosinophils (%), in CS	Eosinophils (%), in SS

With polyps	0.4 (0–74)	0.4 (0–34)
Without polyps	0 (0–38)	0 (0–27)

	*p* = 0.01	*p* = 0.008

CS: cytocentrifuged slides; SS: smeared slides.

## Data Availability

The data used to support the finding of this study (SPSS file) are deposited in the department server of Pisa University Hospital.
